# Impact of SORL1 genetic variations on MRI markers in non-demented elders

**DOI:** 10.18632/oncotarget.9300

**Published:** 2016-05-11

**Authors:** Rui-Hua Yin, Jun Li, Lin Tan, Hui-Fu Wang, Meng-Shan Tan, Wan-Jiang Yu, Chen-Chen Tan, Jin-Tai Yu, Lan Tan, Alzheimer's Disease Neuroimaging Initiative

**Affiliations:** ^1^ Department of Neurology, Qingdao Municipal Hospital, School of Medicine, Qingdao University, Qingdao, China; ^2^ College of Medicine and Pharmaceutics, Ocean University of China, Qingdao, China; ^3^ Department of Radiology, Qingdao Municipal Hospital, School of Medicine, Qingdao University, Qingdao, China

**Keywords:** SORL1, Alzheimer's Disease, Alzheimer's Disease Neuroimaging Initiative (ADNI), hippocampus, parahippocampal, Gerotarget

## Abstract

The sorting protein-related receptor 1 (SORL1 or LR11) gene has been verified to play an important role in the pathologic process of β-amyloid (Aβ) formation and trafficking in Alzheimer's Disease (AD) by plenty of cytological and molecular biological studies. But there were few studies investigated the association of SORL1 gene and neurodegeneration features from a rather macroscopic perspective. In the present study, we explored the effect of SORL1 genotypes on AD-related brain atrophy. We recruited 812 individuals with both baseline and two-year follow-up information from the Alzheimer's Disease Neuroimaging Initiative (ADNI) database and applied multiple linear regression models to examine the association between eight single nucleotide polymorphisms (SNPs) and neuroimaging phenotypes. Finally, four SNPs (rs11219350, rs2298813, rs3781836, rs3824968) showed trend of association with the volume of hippocampus and parahippocampal gyrus but failed to survive the false discovery rate (FDR) correction. Only rs1784933 and rs753780 showed significant association with right parahippocampal gyrus. According to our findings, SORL1 variations influence the atrophy of specific AD-related brain structures, which suggested the potential role of SORL1 in the neurodegeneration of cognitive related regions.

## INTRODUCTION

With a prevalence of 5%-7% in most world regions, dementia has always been a global trouble [1], leading to a great burden for affected individuals, their caregivers, and society [2–4]. Alzheimer's disease (AD), accounts for 50% of all dementia, is a complex neurodegenerative disease [5]. Genetic factors, along with environments components both contribute to the pathogenesis of AD [4, 6]. Over the years, Apolipoprotein E (ApoE) was the only confirmed susceptibility gene for common late onset AD (LOAD; aged more than 65 years) [7, 8]. But gradually, many newly noted genes were found implicated in the risk of AD since the early 1990s. The SORL1—sorting protein-related receptor 1 (also denoted as LR11) is one of the new actors. SORL1 was firstly discovered to be relate to AD in 2004 [9] and the protein level of SORL1 is found decreased in mild cognitive impairment (MCI) and LOAD patients, particularly in the vulnerable areas of the cortex and hippocampus [10]. Subsequently several large genome-wide association studies (GWAS) validated its association with AD [11–15], followed by a series of larger replication studies in both Caucasians [16–22] and Asian [23–26] ethnic lines.

It was hypothesize that the AD biomarker could be categorized into brain β-amyloid (Aβ) plaque deposition (cerebrospinal fluid (CSF) Aβ1-42 and positron emission tomography (PET) Aβ imaging) and neurodegeneration (CSF tau, fluorodeoxyglucose-PET and structural magnetic resonance imaging (MRI)) [27]. Given the current understanding of mechanisms by which SORL1 participate in AD progression, plenty of studies had explored the role of SORL1 in the process of Aβ formation and trafficking and made abundant achievements [28]. But a few researches paid attention to the association of SORL1 and neuroimaging features, while the important role of these neuroimaging biomarkers is becoming more and more prominent. Above all, neuroimaging biomarkers provide a more intuitionistic measurement towards genetic effects on brain formation and function. AD patients generally show disproportionate atrophy in medial, basal, and lateral temporal lobe, and medial parietal cortex on structural MRI [29–31]. Moreover, neuroimaging features can not only assist AD diagnosis but also reflect the AD progression. It is possible that by neuroimaging measures, early slight pathologic change can be detected in MCI or preclinical AD. Besides, the structural MRI is more powerful than CSF tau to predict the future conversion from MCI to AD [32]. Increasing number of studies are suggesting neuroimaging measures can be affected by genetic factors at a great extent, with heritability estimates as high as 80% [33]. ApoE has been substantiated strongly related to neuroimaging biomarkers in AD process [34, 35] and variants of SORL1 were also found to associate with brain MRI and neuropathological measures of neurodegenerative disease in AD [36].

This study was designed to investigate the possible association between SORL1 variations and the neuroimaging endophenotypes of AD. We genotyped multi-loci in SORL1 and explored their associations with specific AD-related brain structures (the hippocampus, the parahippocampal gyrus, the entorhinal cortex, the middle temporal gyrus and the posterior cingulate). We first assess the correlation of SORL1 loci with brain regions in the hybrid group including individuals with normal cognition (NC), MCI and AD. And for further validation, we replicated the analysis in subgroups of NC and MCI within the significant region of interest (ROI). Evidence that the SORL1 genetic risk factors for AD impact on neuroimaging traits would provide crucial support for the involvement of SORL1 genetic variants in AD pathogenesis.

## RESULTS

### Demographic and clinical characteristics of included subjects

The information about the study sample is presented in Table 1. The final dataset comprised 812 individuals, including 281 normal cognitive individuals (NC) (145 women, 74.51±5.56 years), 483 MCI (201 women, 72.28±7.45 years) and 48 AD (18 women, 75.51±9.23 years) at baseline. No significant difference was found on education (*p* = 0.08) among these included subjects (statistical significance criteria *p* < 0.01). As expected, frequency of the ApoE ε4 allele was significantly higher in AD patients than NC and MCI. And compared to NC and MCI subjects, AD patients displayed the worst cognitive scores (*p* < 0.01) on various neuropsychological scales (CDRSB, MMSE, RAVLT, etc.). Furthermore, AD patients showed the most severely atrophy in hippocampus, entorhinal cortex and middle temporal gyrus than MCI and NC groups (*p* < 0.01).

**Table 1:** The demographic and clinical characteristics of the ADNI subjects at baseline

Characteristics	CN group		MCI group		AD group		*P*-value^b^
Age (years)	281	74.51±5.56	483	72.28±7.45	48	75.51±9.23	-
Gender (male/female)	281	136/145	483	282/201	48	30/18	-
Education (years)	281	16.41±2.66	483	15.98±2.82	48	15.73±2.62	0.08
ApoE ε4 (0/1/2)	281	204/70/7	483	262/180/41	48	14/25/9	<0.01
CDRSB (scores)	207	6.54±0.55	406	6.32±0.64	47	5.3±0.72	<0.01
ADAS (scores)	281	29.07±1.15	483	27.89±1.69	48	22.96±2.03	<0.01
MMSE (scores)	281	9.06±4.23	480	15.3±6.65	48	29.8±8.44	<0.01
RAVLT total (scores)	280	44.83±9.6	483	36.16±10.86	47	22.32±7.84	<0.01
FAQ (scores)	281	0.17±0.66	481	2.85±3.99	48	12.6±7.14	<0.01
Hippocampus (mm^3^)	257	7344±895	422	6996±1126	39	5757±948	<0.01
Middle Temporal (mm^3^)	257	20298±2600	422	20186±2735	39	17776±3230	<0.01
Entorhinal (mm^3^)	257	3803±650	422	3610±723	39	2919±705	<0.01
FDG	207	6.55±0.55	406	6.32±0.64	47	5.3±0.72	<0.01

Abbreviation: CN, cognitively normal; MCI, mild cognition impairment; AD, Alzheimer's disease; CDRSB, Clinical Dementia Rating scale sum of boxes; ADAS, Alzheimer's disease Assessment Scale; MMSE, Mini-Mental State Exam; RAVLT, Rey Auditory Verbal Learning Test; FAQ, Functional Activities Questionnaire; FDG, Cerebral Glucose Metabolism Rate measured with fluorodeoxyglucose-positron emission tomography(FDG-PET). *P values for continuous variables are from one-way analysis of variance (ANOVA). P values for categorical data are from chi square test. Data are given as mean ± standard deviation unless otherwise indicate.

### SNP selection and SNP information

We selected SNPs from published GWASs, the meta-analysis and replication studies. After matching the genotype data of ADNI database, eight AD associated SNPs were involved for analysis. Five of them have been validated to associate with AD in GWAS of ethnically distinct populations: rrs11218350, s12364988, rs2298813, rs3824968, rs4935774 [11, 15, 20]. And the other three SNPs (rs3781836, rs1784933, rs753780) were revealed to have intimate relativity with AD, either in neuroimaging characteristics or cognitive impairment [12, 37–39]. Detailed position and function SNP information is showed in Table 2.

**Table 2:** The characteristics of included eight SNPs

SNP	Chr	Position	Minor allele	MAF (Baseline)	H-W (p value) (Baseline)	Previous studied articles (PMID)
rs11218350	11	intron variant	A	0.235	0.1619	18090307
rs12364988	11	exon 6, synonymous codon	C	0.491	0.0091	20413850, 19822782, 23455993, 18938222
rs1784933	11	intron variant	G	0.073	0.5164	25450149
rs2298813	11	missense, utr variant 5 prime	A	0.054	0.0511	20413850, 24938503, 25382023
rs3781836	11	intron variant	A	0.123	0.6385	23455993
rs3824968	11	exon 34, synonymous codon	A	0.306	0.1336	20413850, 20625269, 18063222, 18938222, 19368828, 19584446, 20667857, 21997402, 24083537, 25659857
rs4935774	11	intron variant, upstream variant 2KB	C	0.248	0.3542	24938503
rs753780	11	intron variant	T	0.099	0.3154	19125160, 23318115

**Abbreviation:** Chr, Chromosome; MAF, Minor Allele Frequency; SNP, Single Nucleotide Polymorphism; H-W, Hardy-Weinberg balance.

### Impacts of SORL1 genotypes on MRI measures in hybrid group

At baseline, we found no significant association (Pc < 0.05) between the SORL1 loci and these ROIs. Only A allele of rs3781836 showed trend to suppress the atrophy of left hippocampus (*p* = 0.04342) and A allele of rs2298813 showed trend to activate the atrophy of parahippocampal gyrus (*p* = 0.0226). But all these founding failed in the FDR correction (Supplementary Table 1). And both of these two loci failed to show association with AD in the large European descent meta-analysis.

**Figure 1:**
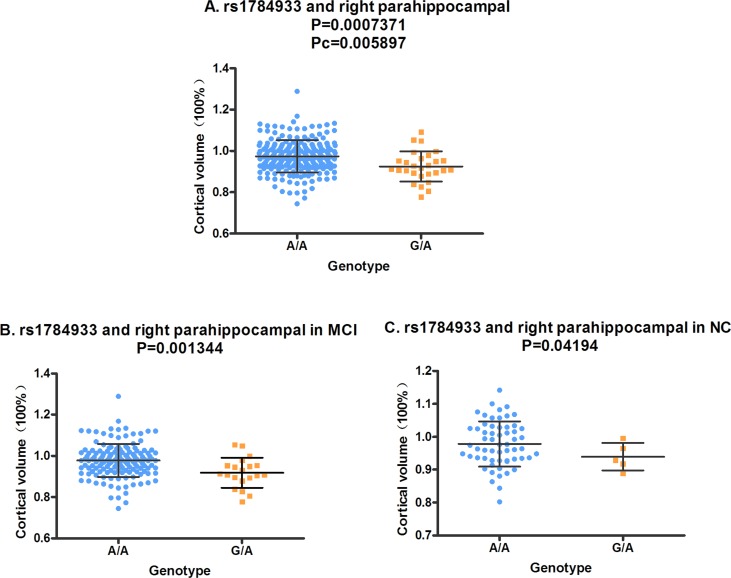
The correlation between rs1784933 and right parahippocampal volume in the two-year follow-up study **A.** Rs1784933 was associated with the volume of right parahippocampal in hybrid population. **B.** Rs1784933 was associated with the volume of right parahippocampal in MCI group. **C.** Rs1784933 was associated with the volume of right parahippocampal in NC group.

**Figure 2:**
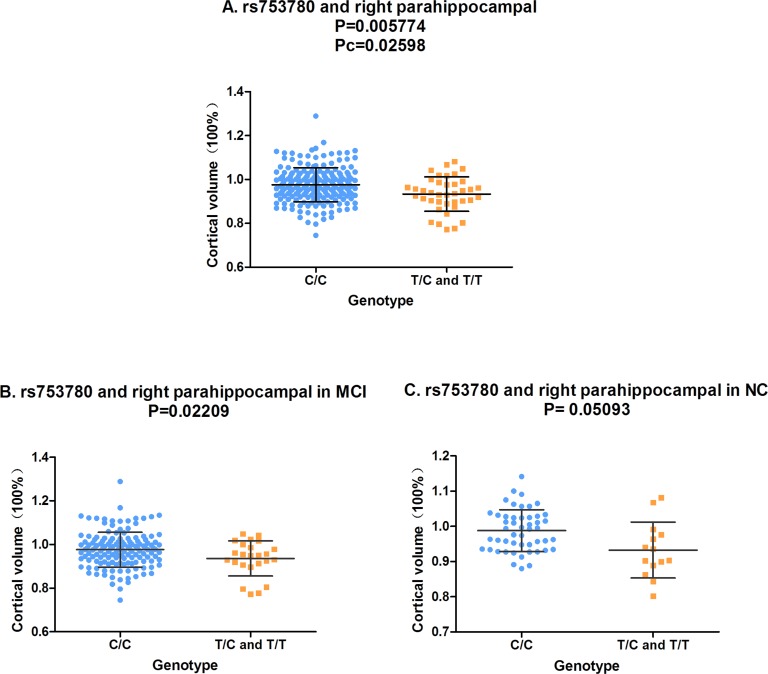
The correlation between rs753780 and right parahippocampal volume **A.** Rs753780 was associated with the volume of right parahippocampal in hybrid population. **B.** Rs753780 was associated with the volume of right parahippocampal in MCI group. **C.** Rs753780 showed no association with the volume of right parahippocampal in NC group.

In the analysis of the two-year follow-up study, we used the volume ratio of two-year follow-up to baseline as the calculated value. Still no significant association (Pc < 0.05) was found between the SORL1 loci and hippocampus volume. Although the variants of rs11218350 (*p* = 0.0345, left) and rs3824968 (*p* = 0.03061, right) showed trend to decrease the atrophy rate of the hippocampus, none of these differences achieved the significant level in the FDR test. And in the meta-analysis of 74 046 participants, only rs11218350 was revealed to link to AD (*p* = 0.0065). The variants of rs1784933 and rs753780 were found to increase the atrophy rate of the parahippocampal gyrus in the two-year follow-up study. But both of these two SNPs were identified to be not associated with AD in the large-scale meta-analysis. Rs1784933 showed remarkable association with the atrophy of bilateral parahippocampal gyrus but only the relationship with the right parahippocampus survive the FDR correction (right: *p* = 0.0007371 and Pc = 0.005897; left: *p* = 0.04121, Pc = 0.2452) (Figure 1-A). The carrier of the heterozygosis mutation of rs1784933 (G/A) showed a higher atrophy rate (7.5%) than wild homozygous (A/A) (2%) in right parahippocampal gyrus. Rs753780 associated with the atrophy rate of right parahippocampus with a P value of 0.005774 and survived the FDR correction (Pc = 0.0231) (Figure 2-A). The mutation homozygote of rs753780 (T/T) showed a higher atrophy rate (7.5%) than the carrier of the heterozygosis mutation (6.1%) and wild homozygous (C/C) (2%) in right parahippocampal gyrus.

### Validation of the association between SORL1 genotypes and MRI measures in subgroups

In the analysis of hybrid population, variants of rs1784933 and rs753780 were found to increase the atrophy rate of right parahippocampal gyrus in two-year longitudinal study. For further validation, we selected the parahippocampal gyrus as our sole ROI and tested its association with rs1784933 and rs753780 in MCI and NC sub-groups of two-year longitudinal study. The association of rs1784933 and right parahippocampal gyrus was replicated in the MCI (*p* = 0.001344) and NC (*p* = 0.04194) group (Figure 1-B and Figure 1-C). Moreover variants of rs1784933 were also found to increase the atrophy rate of left parahippocampal gyrus with a P value of 0.0255 in MCI group. The results of rs753780 related to right parahippocampal gyrus was only replicated in MCI group (*p* = 0.02209) (Figure 2-B).

## DISCUSSION

Our results showed that the mutant genotypes of rs1784933 and rs753780 significantly activate the atrophy of right parahippocampal gyrus. These finding suggested the relevance between the SORL1 genetic variations and the neuroimaging biomarkers, indicating the involvement of SORL1 in neurodegeneration in AD.

It is widely accepted that the Aβ biomarker abnormalities precede neurodegenerative biomarker abnormalities [27]. In this case, the SORL1-related MRI atrophy is probable the downstream consequence of the Aβ abnormalities. In the replication study, we found that SORL1 genetic variations were more inclined to be associated with atrophy rate in MCI individuals. The brain atrophy attributes to the stable normal aging and the changeable abnormal pathological insults. Without question in stage of MCI, pathological insults played a more prominent role in brain atrophy. So it can be supposed that the potential pathways of SORL1 may be associated with pathological insults of Aβ abnormalities. But we cannot exclude the other pathways by which SORL1 directly or indirectly act on the neural injury leading to the cerebral atrophy.

The dominant role of the histopathological changes of hippocampus in AD has been widely acknowledged [40] and there has been studies suggesting the diagnostic significance of MR-based hippocampal volumetry [41, 42]. According to our results, A allele of rs3781836 showed protective inclination on hippocampus at baseline and A allele of rs11218350 and rs3824968 showed protective trend on hippocampus in two-year follow-up study. The decreasing sample size (from 500+ to 200+ after two-year follow-up) in the current study is a possible contributor. Moreover, the prime factors contributing to hippocampal atrophy rate (HAR) includes age, gender, ApoE ε4 status, intracranial volume, white matter lesions and Aβ levels, and Aβ status is a significant predictor of HAR [43]. However in our multiple linear regression analysis, we did not include white matter lesions and Aβ levels as collaborators variables. This deficiency might also affect our results of hippocampus volume greatly.

The atrophy of parahippocampal gyrus was proposed to serve as an early biomarker of AD and confirmed to discriminate better than hippocampal volume, especially in the early phase of AD [44–46]. In the present study, we found variants of rs1784933 and rs753780 significantly activate the atrophy of right parahippocampal gyrus. Previous studies have found the right parahippocampal gyrus showed imbalanced bidirectional effective connections in the default mode network (DMN) and hippocampus network. Along with our findings as the morphological evidence, it may provide neurophysiological explanations for AD patients’ memory impairment during the encoding processes [47]. Besides, it should be noted that the majority of our positive results were inclined to the right side. Undoubtedly, most of our subjects are dextromanuality and the right hemicerebrum is the non-dominant hemisphere. It is worth to consider whether the dominant hemisphere is equipped with stronger pathological-resistant ability to avoid excessive volume change and further researches are wanted to validate this hypothesis.

We assessed the genetic risk with imaging measures as quantitative traits (QTs) or continuous phenotypes in order to increase statistical power and decrease sample size requirements, which have advantages over traditional case-control designs. But the various neuroimaging data were available only in half of participants with MRI information and then the sample size had a relative reduction. Moreover, the ADNI dataset was limited to Caucasians to avoid genetics stratification across ethnicities, but the 9 loci in SORL1 have various frequencies in different races. This contradiction determines the racial limitation of our research and the replications in other races are necessary. For another, our study just provided evidence for association between SORL1 genetic variants with MRI neuroimaging traits, but we cannot illustrate the mechanistic means by which they may influence expression levels or protein structures or how they affect phenotypes. Recently, researchers used hIPSCs to examine the possible contributions of SORL1 genetic variation to sporadic AD-related phenotypes and found that human neurons carrying SORL1 variants with an increased AD risk show a reduced response to treatment with brain derived neurotrophic factor (BDNF), at the level of both SORL1 expression and APP processing [48]. This might show us a brilliant prospect for further mechanistic research.

In conclusion, the current study investigated the effect of common variations at the SORL1 locus on neuroimaging phenotypes in hybrid population. And the main findings that SORL1 genotypes were associated with the notable AD-related brain structures, provided evidence supporting the hypothesis that SORL1 genetic variations modulate the alteration of the biomarkers of neuronal degeneration. Future studies for ethnic diversity and detailed mechanism are required to expand these findings.

## MATERIALS AND METHODS

### ADNI dataset

All data used in this study was obtained from the ADNI database. The ADNI is a large, multisite, longitudinal collaborative study, which was launched in 2003 by the National Institute on Aging (NIA), the National Institute of Biomedical Imaging and Bioengineering (NIBIB), the Food and Drug Administration (FDA), private pharmaceutical companies, and nonprofit organizations (http://www.adni-info.org) [49, 50]. It aims at testing whether serial MRI, PET, other biological markers, as well as clinical and neuropsychological assessment can be combined to measure the progression of MCI and early AD. After 3 protocols (the initial ADNI-1, followed by ADNI-GO and ADNI-2), the ADNI have recruited over 1,500 adults from more than 50 sites across the United States and Canada, aging from 55 to 90 years, including cognitively normal individuals, MCI and early AD patients [51]. All ADNI studies were conducted according to the Good Clinical Practice guidelines, the Declaration of Helsinki, and the U.S. 21 CFR Part 50 (Protection of Human subjects) and Part 56 (Institutional Review Boards). The present study was approved by the Institutional Review Boards of all participating sites and written informed consent was obtained from all participants or authorized representatives before the study.

### Participants

Participants were screened and enrolled according to criteria outlined in the ADNI study protocol (http://www.adni-info.org/scientists/adnistudyprocedures.aspx). We restricted the participants to whose genotype data of SORL1 SNPs were available and comprised 812 individuals. Baseline and longitudinal data of structural MRI results were collected and all participants underwent a battery of clinical tests including Clinical Dementia Rating scale sum of boxes (CDRSB), Alzheimer's disease Assessment Scale (ADAS-cog), Mini-Mental State Exam (MMSE), Rey Auditory Verbal Learning Test (RAVLT) and Functional Activities Questionnaire (FAQ) at baseline. According to the National Institute of Neurological and Communication Disorders/Alzheimer's Disease and Related Disorders Association criteria for probable AD (NINCDS-ADRDA: probable AD), participants of AD was included if with a MMSE score between 20 and 26, a global Clinical Dementia Rating (CDR) of 0.5 or 1.0 and a CDRSB of 1.0 to 9.0. Amnestic MCI subjects fulfilled a MMSE score of 24 to 30 as well as a CDR score of 0.5 and cognitively normal control individuals with a CDR score of 0. In addition, subjects with any serious neurological disease except for possible AD, any history of brain lesions or trauma, or psychoactive medication use (including antidepressants, neuroleptics, chronic anxiolytics, or sedative hypnotics) were not be included in this study.

### Genotype data

We extracted the SNP genotypes of SORL1 from the PLINK format data of Genome-wide association study (GWAS) in ADNI database. The ADNI applied the Illumina Infinium Human610-Quad Bead Chip (Illumina, Inc., San Diego, CA) including 620,901 SNP and CNV markers to conduct genotyping for GWAS data, with ADNI-2/GO participants using Illumina Human Omni Express Bead Chip [52]. The quality control (QC) procedures were performed using PLINK version 1.07 (http://pngu.mgh.harvard.edu/~purcell/plink/), including filters for missingness, heterozygosity, and concordance between genotype-determined and reported sex. The inclusion criteria are as follows: minimum call rates > 90%, minimum minor allele frequencies (MAF) > 0.01 and Hardy-Weinberg equilibrium test *p* > 0.001.

### Neuroimaging

We used the high quality data of structural volumetric MRI, which were available in the ADNI data archive provided by the University of California, San Francisco (UCSF) medical center, to conduct association test of SORL1 genotypes with brain structure. The raw Digital Imaging and Communications in Medicine images were downloaded from the public ADNI site (http://www.loni.ucla.edu/ADNI/Data/index.shtml) and parameter values were available at http://www.loni.ucla.edu/ADNI/Research/Cores/. All MRIs were processed using the FreeSurfer version 5.1 (http://surfer.nmr.mgh.harvard.edu/) based on the 2010 Desikan-Killany atlas [53, 54]. The procedure included averaging of multiple volumetric T1 weighted images, intensity normalization, removal of non-brain tissue, segmentation of the subcortical white matter and deep gray matter volumetric structures [55], automated Talairach transformation, tessellation of the gray matter white matter boundary, automated topology correction, and surface deformation following intensity gradients to optimally locate the gray-white and gray-cerebrospinal fluid boundary where the greatest shift in intensity defines the transition to the other tissue class [56]. In addition, cortical thickness measurements were then obtained by calculation of the distance between the white and grey matter surfaces at each point across the entire cortical surface (per hemisphere) [57]. We used the ROIs strategies in MRI analysis to assess the relationship between SORL1 and AD and focused on the discriminant brain regions that had been found to be strongly associated with AD by previous studies: hippocampus, parahippocampal gyrus, middle temporal gyrus, posterior cingulate and the entorhinal cortex.

### Statistical analysis

We used an additive model for genotype data analysis and each of the eight SNPs was examined for associations with the neuroimaging phenotypes. Differences in continuous variables were inspected using one-way analysis of variance (ANOVA), while categorical data were tested using chi-square test. In addition, a multiple linear regression model which considered age, gender, education, and ApoE ε4 status as covariates was applied in analyses of neuroimaging measures to test possible correlation between SORL1 genotypes and various phenotypes. We examined the association between SORL1 variations and AD-related brain atrophy in baseline condition and the two-year follow-up state respectively in hybrid population. It is noted that to eliminate the possible bias caused by individual cerebral volume difference, we used the atrophy ratio of two-year follow-up to baseline as the calculated value of volumes for longitudinal analysis. And we verified the correlations between of these new positive variants and AD susceptibility in a large-scale European descent dataset from a meta-analysis of AD GWAS, including 74 046 individuals [58]. After the study in the hybrid population, we replicated the positive results in subgroup (MCI and NC) for validation (AD was excluded for constrained sample size). All these statistical analyses were performed by R 3.12 (http://www.r-project.org/) and PLINK version 1.07. To control for multiple hypothesis testing, we used the false discovery rate (FDR) for correction [59] and statistical significance was defined for FDR-corrected *p* < 0.05.

## SUPPLEMENTARY MATERIALS TABLES


