# Supplementary material for: Impact of SORL1 genetic variations on MRI markers in non-demented elders
Source: Oncotarget. 2016 May 11;7(22):31689–98. doi: 10.18632/oncotarget.9300 (PMC5077969; doi:10.18632/oncotarget.9300)
Supplement: Supplementary file 1 [file oncotarget-07-31689-s001.pdf]

Impact of SORL1 genetic variations on MRI markers in non-demented elders

Supplementary Material

Supplementary Table 1. Associations of SORL1 loci with brain regions on MRI in hybrid population

ROI	SNPs	Baseline						Follow-up (2 year)				
		Sample	P-value		Pc-value			Sample	P-value		Pc-value	
			Left	Right	Left	Right			Left	Right	Left	Right
Hippocampus (Volume)	rs11218350	536	0.9297	0.8273	0.9297	0.8745		238	**0.0345**	0.2286	0.276	0.8715
	rs12364988	536	0.5795	0.5486	0.7727	0.7314		238	0.4182	0.944	0.6152	0.9972
	rs1784933	536	0.1459	0.0645	0.389	0.3432		238	0.4614	0.6451	0.6152	0.91
	rs2298813	535	0.09339	0.1116	0.3736	0.3432		238	0.6222	0.9972	0.6648	0.9972
	rs3781836	536	**0.04342**	0.3243	0.3474	0.6486		238	0.2495	0.6825	0.5427	0.91
	rs3824968	535	0.3758	0.4779	0.7385	0.7314		238	0.08163	**0.03061**	0.3265	0.2449
	rs4935774	533	0.4615	0.1287	0.7385	0.3432		237	0.6648	0.6174	0.6648	0.91
	rs753780	535	0.834	0.8745	0.9297	0.8745		237	0.2713	0.3268	0.5427	0.8715
Parahippocampal (Volume)	rs11218350	536	0.7074	0.7594	0.7074	0.9124		237	0.6826	0.5945	0.9101	0.988
	rs12364988	536	0.4111	0.9124	0.7074	0.9124		237	0.992	0.8649	0.992	0.988
	rs1784933	536	0.5331	0.7902	0.7074	0.9124		237	**0.04121**	**0.0007371**	0.2452	**0.005897**
	rs2298813	535	0.1495	**0.0226**	0.5978	0.1808		237	0.06131	0.988	0.2452	0.988
	rs3781836	536	0.7026	0.9035	0.7074	0.9124		237	0.8632	0.1126	0.9865	0.3003
	rs3824968	535	0.5572	0.1738	0.7074	0.6613		237	0.3973	0.7274	0.6357	0.988
	rs4935774	533	0.1171	0.248	0.5978	0.6613		236	0.2501	0.9582	0.5286	0.988
	rs753780	535	0.531	0.6854	0.7074	0.9124		236	0.2643	**0.005774**	0.5286	**0.0231**

**Middle Temporal (volume)**	rs11218350	536	0.8074	0.8672	0.9873	0.8672	238	0.5601	0.6308	0.8647	0.721
	rs12364988	536	0.8376	0.2367	0.9873	0.3788	238	0.2949	0.4501	0.6535	0.6849
	rs1784933	536	0.6059	0.3519	0.9873	0.4022	238	0.6485	0.5136	0.8647	0.6849
	rs2298813	535	0.08943	0.1668	0.7154	0.3788	238	0.3229	0.1221	0.6535	0.3751
	rs3781836	536	0.2887	0.07894	0.9873	0.3788	238	0.7624	0.2525	0.8713	0.505
	rs3824968	535	0.7054	0.2021	0.9873	0.3788	238	0.3267	0.8278	0.6535	0.8278
	rs4935774	533	0.9873	0.3209	0.9873	0.4022	237	0.9925	0.1407	0.9925	0.3751
	rs753780	535	0.958	0.1191	0.9873	0.3788	237	0.2577	**0.04399**	0.6535	0.3519
**Posterior Cingulate (volume)**	rs11218350	536	0.2716	0.1914	0.7241	0.7655	238	0.7451	0.811	0.9018	0.811
	rs12364988	536	0.5286	0.6628	0.8012	0.8165	238	0.6134	0.6276	0.9018	0.7436
	rs1784933	536	0.0533	0.7318	0.4264	0.8165	238	0.1488	0.5385	0.9018	0.7436
	rs2298813	535	0.7749	0.7538	0.8012	0.8165	238	0.5622	0.09505	0.9018	0.4392
	rs3781836	536	0.2431	0.1803	0.7241	0.7655	238	0.8149	0.5787	0.9018	0.7436
	rs3824968	535	0.6837	0.4345	0.8012	0.8165	238	0.7287	0.488	0.9018	0.7436
	rs4935774	533	0.4174	0.4061	0.8012	0.8165	237	0.9018	0.6506	0.9018	0.7436
	rs753780	535	0.8012	0.8165	0.8012	0.8165	237	0.2621	0.1098	0.9018	0.4392
**Entorhinal Cortex (Thickness)**	rs11218350	536	0.3751	0.6644	0.695	0.7593	238	0.5119	0.5496	0.6189	0.6403
	rs12364988	536	0.4764	0.5451	0.695	0.7268	238	0.4449	0.4002	0.6189	0.6403
	rs1784933	536	0.5212	0.5362	0.695	0.7268	238	0.9186	0.5983	0.9186	0.6403
	rs2298813	535	0.4793	0.2264	0.695	0.6038	238	0.09837	0.5322	0.6189	0.6403
	rs3781836	536	0.7825	0.0837	0.8943	0.556	238	0.4114	0.6403	0.6189	0.6403
	rs3824968	535	0.9775	0.8877	0.9775	0.8877	238	0.2386	0.1164	0.6189	0.4655
	rs4935774	533	0.4631	0.3264	0.695	0.6528	237	0.1795	0.6142	0.6189	0.6403
	rs753780	535	0.252	0.139	0.695	0.556	237	0.5416	0.06064	0.6189	0.4655

Supplementary Table 2. Significant results from multiple linear regression analysis for each genotype

Neuroimaging phenotypes	ROI	SNPs	Sample	Means±SD of variables			P-value	Pc-value
				Minor allele homozygotes	Heterozygous	Major allele homozygotes		
Volume	Right Parahippocampal	rs1784933	238	G/G(NA±NA)	G/A(0.9252±0.07056)	A/A(0.9806±0.08028)	0.0007371	0.005897
	Right Parahippocampal	rs753780	237	T/T(0.9256±0.02371)	T/C(0.9391±0.0827)	C/C(0.9805±0.07995)	0.005774	0.0231

Supplementary table 3 The replicated results of the associations of *SORL1* loci with parahippocampal gyrus volume in subgroup population

Phenotypes	SNP	Subgroup	Sample	P-value	
				Left	Right
The volume of parahippocampal gyrus	rs1784933	MCI	168	**0.0255**	**0.001344**
		NC	60	0.5952	**0.04194**
	rs753780	MCI	168	0.2419	**0.02209**
		NC	59	0.9442	0.05093
